# Genome scans for divergent selection in natural populations of the widespread hardwood species *Eucalyptus grandis* (Myrtaceae) using microsatellites

**DOI:** 10.1038/srep34941

**Published:** 2016-10-17

**Authors:** Zhijiao Song, Miaomiao Zhang, Fagen Li, Qijie Weng, Chanpin Zhou, Mei Li, Jie Li, Huanhua Huang, Xiaoyong Mo, Siming Gan

**Affiliations:** 1 State Key Laboratory of Tree Genetics and Breeding, Chinese Academy of Forestry, Xiangshan Road, Beijing 100091, China; 2 Key Laboratory of State Forestry Administration on Tropical Forestry Research, Research Institute of Tropical Forestry, Chinese Academy of Forestry, Longdong, Guangzhou 510520, China; 3 Baoshan University, Yuanzheng Road, Baoshan 678000, China; 4 College of Forestry, South China Agricultural University, 284 Block, Wushan Street, Guangzhou 510642, China; 5 Guangdong Academy of Forestry, Longdong, Guangzhou 510520, China

## Abstract

Identification of loci or genes under natural selection is important for both understanding the genetic basis of local adaptation and practical applications, and genome scans provide a powerful means for such identification purposes. In this study, genome-wide simple sequence repeats markers (SSRs) were used to scan for molecular footprints of divergent selection in *Eucalyptus grandis*, a hardwood species occurring widely in costal areas from 32° S to 16° S in Australia. High population diversity levels and weak population structure were detected with putatively neutral genomic SSRs. Using three *F*_ST_ outlier detection methods, a total of 58 outlying SSRs were collectively identified as loci under divergent selection against three non-correlated climatic variables, namely, mean annual temperature, isothermality and annual precipitation. Using a spatial analysis method, nine significant associations were revealed between *F*_ST_ outlier allele frequencies and climatic variables, involving seven alleles from five SSR loci. Of the five significant SSRs, two (EUCeSSR1044 and Embra394) contained alleles of putative genes with known functional importance for response to climatic factors. Our study presents critical information on the population diversity and structure of the important woody species *E. grandis* and provides insight into the adaptive responses of perennial trees to climatic variations.

Natural populations may survive climate change through migration and local adaptation^1^,^2^. In sessile plants, while migration involves propagule (i.e. seed and plant fragment) dispersal and population establishment in new locations^2^, local adaptation represents higher fitness of local than nonlocal populations resulting from divergent selection among environments^3^. As adaptation is often inherent in and interplayed with migration^2^, migration can not be viewed simply as an alternative to occurrence of local adaptation^4^. With the occurrence of rapid climate change, the migration rates observed commonly (e.g. 20–40 km per century) are far below the rates projected (e.g. 300–500 km per century) to track future climate shifts^1^, and local adaptation to future climatic conditions will likely be necessary for long-term *in situ* population persistence^5^.

Divergent natural selection, as exerted usually by environmental gradients, is the major driving force of local adaptation. Such selection can result in gene frequency changes among populations within a species, ultimately leading to variation in both phenotypic traits and genetic structure even in the presence of gene flow^6^,^7^. Identifying the effects of loci or genes under natural selection is important for elucidating the genetic basis of adaptation to different environments and also for practical applications in biodiversity conservation and selective breeding^8^,^9^. Using a candidate gene or genome scan approach, genomic loci associated with natural selection could be identified through linkage disequilibrium (LD) and quantitative trait locus mapping of adaptive traits, detection of between-population differentiation (*F*_ST_) outlier loci and analysis of alleles that are correlated with environmental variables^8^,^10^.

Genome scans provide a powerful means in population genetics studies to detect outlier loci putatively under selection^11^,^12^ and identify alleles associated with environmental variables^13^. In contrast to a candidate gene approach, in which a limited set of candidate genes potentially influencing the adaptive trait are used, genome scans investigate a large number of loci throughout the genome even in the absence of phenotypic information^14^. To date, genome scans have been extensively employed for detecting loci or genes under selection in model organisms (e.g. *Zea mays* L.^15^), but its potential still remains to be explored for most non-model species^10^,^16^. Moreover, while most genome scan studies on natural selection have used high-throughput array-based single nucleotide polymorphism markers, in which the finite number of markers (at least in non-model species) and the marker nature of bi-allelism can limit the genotyping of all variations at a genomic region, few studies have attempted to use multi-allelic markers, such as microsatellites (or simple sequence repeats, SSR).

The broadleaved tree genus *Eucalyptus* L’Hér. (family Myrtaceae) encompasses more than 780 species and subspecies native to the Australian continent and surrounding islands^17^. It is highly diverse and displays significant adaptability and phenotypic plasticity^18^. With the projection of pronounced temperature warming and rainfall decline in Australia, eucalypts are particularly interesting for climate change studies, considering their poor dispersal capability and limited gene flow^19^. Also, *Eucalyptus* species and hybrids constitute the most widely planted hardwood trees in the world (plantations >20 million ha, http://git-forestry.com/Global_Eucalyptus_Map.htm) and are therefore important for the global forest-related industries. Within *Eucalyptus*, *E. grandis* Hill ex Maiden (flooded gum or rose gum) is one of the most important species in terms of breeding and genomics efforts^18^ and has been selected as the second tree species (only after *Populus trichocarpa* Torr. & Gray) for whole genome sequencing^20^. *E. grandis* is a diploid species with 2n = 22 chromosomes and 640 Mb genome size^20^. It is distributed naturally mainly in coastal areas from northern New South Wales (32° S in latitude) to southern Queensland (26° S) in Australia, with two outlier patches in central (22° S) and northern (16−18° S) Queensland. Its natural forests are mostly on low lands and hills ranging from sea level to ~600 m in altitude^21^. Of note, *E. grandis* displays significant variation in adaptive phenotypic traits, such as growth and frost tolerance (e.g. Rockwood & Meskimen^22^). A relatively low level of population differentiation has been revealed in *E. grandis* by isozyme markers (*G*_ST_ = 0.12)^23^, suggesting a weak population structure. Thus far, genomic locus-association analyses have detected loci with significant effects on economic traits in *E. grandis* and other *Eucalyptus* species^18^. However, there have been no association studies specifically aiming at local adaptation in *E. grandis*. In addition, even in *Eucalyptus*, only two studies have recently reported candidate loci for diversifying selection in natural populations of *E. gomphocephala* DC.^24^ and *E. tricarpa* (L.A.S. Johnson) L.A.S. Johnson & K.D. Hill^25^.

In this study, we used 110 SSR markers distributed across the genome sequence of *E. grandis*, including 45 genomic SSR (gSSR) and 65 expressed sequence tag (EST) derived SSR (EST-SSR) markers, to search for molecular footprints of divergent selection in *E. grandis* natural populations. The genetic diversity and population structure at putatively neutral gSSR loci were first assessed for 16 *E. grandis* populations across the species range. The *F*_ST_ outlier SSRs were then tested against the 110 SSRs to identify loci potentially involved in divergent natural selection. SSR alleles associated with climatic variables were finally investigated to reveal gene polymorphisms contributing to local adaptation to temperature, isothermality and/or precipitation.

## Materials and Methods

### Population sampling and DNA extraction

**Figure 1:**
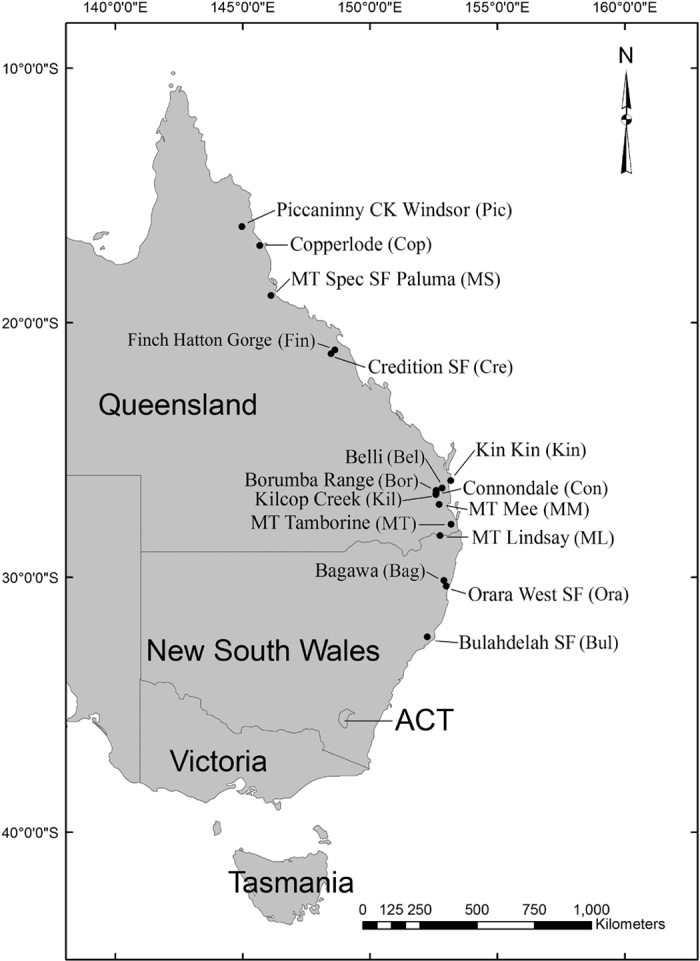
Geographic distribution of the 16 *Eucalyptus grandis* populations studied. The map was generated using software ArcGIS 10.0 (http://www.esri.com/software/arcgis/). Full description of the populations can be found in Table 1. SF, state forest; ACT, Australian Capital Territory.

A total of 159 individuals were sampled from 16 provenances across the range of *E. grandis* (Fig. 1 and Table 1), each provenance representing one population and each individual originating from an open-pollinated mother tree in the native forests. The sample size ranged from nine to twelve individuals per population (Table 1). The mother trees were at least 100 m apart to minimise the risk of collecting seed from closely related trees (R Arnold, Australian Tree Seed Centre, Australia, pers. comm.). The original provenance seedlots had been provided by Australian Tree Seed Centre (Canberra, Australia). The leaf samples were stored at −80 °C prior to DNA extraction. Leaf samples were collected in July 2011 from a 2-year-old *E. grandis* provenance-family trial located at Zhaoqing City (112°27′ E, 23°03′ N), Guangdong Province, China.

Leaf tissues (~300 mg) were powder homogenized in a MM400 mixer mill (Retsch GmbH, Haan, Germany), and genomic DNA was subsequently extracted using a modified CTAB method^26^. DNA concentration and quality were determined by agarose gel electrophoresis and a Nanodrop 2000 spectrophotometer (Thermo Fisher Scientific Inc., Waltham, MA, USA).

### Microsatellite markers and their genotyping

In total, 110 SSR markers distributed across the 11 main scaffolds and one small scaffold (Supplementary Table S1) were used in this study, including 45 gSSRs^27^,^28^ and 65 EST-SSRs^29^,^30^,^31^. Polymerase chain reactions (PCRs) were performed in a 10 μL volume on a DNA Engine (Bio-Rad Laboratories, Hercules, CA, USA) following a florescent-dUTP based protocol^32^, with MgCl_2_ concentration and melting temperature (*T*_m_) depending on marker. The PCR products (1 μL) were diluted 1:10.5 with loading buffer (9.34 μL deionized formamide and 0.16 μL GeneScan 500LIZ) and then detected on an ABI 3130xl genetic analyzer using GeneMapper 4.1 software (Applied Biosystems, Foster City, CA, USA). Sixteen individuals, each randomly selected from one population, were PCR repeated to test the marker reproducibility.

### Marker polymorphism, population diversity and structure

For each locus, number of alleles (*N*_A_), observed heterozygosity (*H*_O_), expected heterozygosity (*H*_E_) and fixation index (*F*) were estimated with GENALEX 6.4.1^33^. Polymorphic information content (PIC) was computed with PowerMarker 3.25^34^. Null allele frequencies (NAFs) were assessed following 50,000 bootstrap resamples in FREENA^35^. Hardy-Weinberg equilibrium (HWE, with manual Bonferroni correction for *P* values) was tested over all populations with GENEPOP 4.2^36^. The software FSTAT 2.9.3.2 (http://www2.unil.ch/popgen/softwares/fstat.htm) was used to investigate per-locus inbreeding coefficients of individuals relative to the total population (*F*_IT_) and to the sub-population (*F*_IS_), *F*_ST_ and allelic richness (*A*_R_, based on the minimal sample size of nine individuals) as well as between-locus LD (with Bonferroni correction).

Only the genomic SSRs that neither departed significantly from HWE (*P* < 0.01) nor showed *F*_ST_ outlying values in lositan analysis on 16 populations (implemented as stated below) were included (31 markers, see Results) in subsequent population diversity and structure analyses. For each population, average number of alleles per locus (*A*_NA_), *H*_O_, *H*_E_, *F* and *A*_R_ were estimated similarly as above. Significance in diversity differences between populations or population groups was tested using the ‘Compare among groups of samples’ function with ‘two sided’ option and 1,000 permutations in fstat 2.9.3.2 (http://www2.unil.ch/popgen/softwares/fstat.htm). Nei’s genetic distance between populations was calculated for construction of an unweighted pair group method with arithmetic mean (UPGMA) dendrogram in POWERMARKER 3.25^34^. The package GENALEX 6.4.1^33^ was used under default settings to conduct analyses of principal coordinates (PCoA), overall between-population differentiations (*F*_ST_) and molecular variances (AMOVA). The Bayesian clustering program STRUCTURE 2.3.4^37^ was performed to assign individuals to a number (*K*) of genetically homogeneous clusters assuming an admixture model and correlated allele frequencies between populations. For each of *K* values (1−16), the Markov Chain Monte Carlo (MCMC) sampling was replicated with 10 iterations^38^ each following 100,000 burn-ins and 100,000 MCMC repetitions. The optimal *K* value was determined by the highest Δ*K* method^39^ in STRUCTURE HARVESTER 0.6^40^ and by a complementary assessment of the change in lnP(*K*) slope with increase in each *K* value.

### Climatic data and partitioning of populations

Values of 19 climatic variables during the years 1950–2000 were obtained for each original population location from a standard set of climate grids (http://www.worldclim.org/) at a spatial resolution of 30 arc-seconds^41^. Correlations between the climatic variables were computed using R function COR.TEST, and only three non-correlated variables (mean annual temperature, isothermality and annual precipitation, see Results) were included in subsequent association analysis of candidate loci.

The 16 populations were partitioned into homogeneous groups using a *k*-means analysis on each of the non-correlated climatic variables^42^, as implemented similarly in black spruce [*Picea mariana* (Mill.) B.S.P.]^43^.

### Detection of *F*_ST_ outlier loci

Three *F*_ST_ outlier detection methods were used to test all the 110 SSR loci for evidence of natural selection among the population groups of each climatic partition. The first was a summary-statistics method^44^ implemented in LOSITAN^45^. The outlying values of *F*_ST_ were identified from a plot of *F*_ST_ vs. expected heterozygosity. Following the infinite allele mutation model, the distribution of *F*_ST_ vs. expected heterozygosity was calculated in LOSITAN with 100,000 simulation replicates under the option of neutral mean *F*_ST_. Markers outside the 95% and 5% confidence intervals of the distribution were considered as candidates (outliers) for positive (divergent) and balancing selections, respectively. False discovery rate (FDR) was set to 0.01.

The second was also a summary-statistics method^44^ modified for hierarchically structured populations^46^ as implemented in ARLEQUIN 3.5^47^. As the hierarchical structure was considered, coalescent simulations (500,000) were implemented under a hierarchical island model to obtain more realistic null distribution for *F*_ST_ statistics and therefore avoid possible false positives. Significant loci at the 95% or 5% confidence level were recognized as candidates for positive (divergent) selection or balancing selection, respectively.

The third was a hierarchical Bayesian modeling method modified from Beaumont and Balding^48^ and implemented in BAYESCAN 2.1^49^. Three runs were performed in BAYESCAN with standard parameters, except 100 prior odds for the neutral model. FDR was set to 0.01. A Bayesian factor and its logarithm value were generated for each marker and thereafter classified into five grades according to Jeffrey’s scale of evidence of selection (http://cmpg.unibe.ch/software/bayescan/index.html), namely, barely worth mentioning, substantial, strong, very strong and decisive evidences, in which the last three grades were considered as candidates for divergent selection.

### Detection of candidate SSR loci associated with environmental variables

The *F*_ST_ outlier loci detected above were corroborated using a spatial analysis method (SAM)^13^. The associations between allele frequencies and climatic variables were tested using the likelihood ratio (G) and Wald tests^13^. With the Bonferroni correction, the significance was declared at 99.99% confidence level, and a conservative alpha of 0.01 was adopted in the Wald test to reduce false positives. Moreover, significant associations were further evaluated in OFFICE EXCEL 2010 (Microsoft Corp., Redmond, WA, USA) to correct allele frequency autocorrelation using univariate linear regressions between group allele frequencies and environmental variables.

## Results

### Marker polymorphism, population diversity and structure

In total, 1,857 alleles were identified at the 110 polymorphic SSR loci. Single-locus parameters exhibited striking differences among loci (Supplementary Table S2), with *N*_A_ ranging from three to 52 (mean 16.9), *H*_O_ from 0.209 to 1.000 (mean 0.623), *H*_E_ from 0.200 to 0.890 (mean 0.706), *A*_R_ from 1.434 to 3.835 (mean 3.027) and NAF from zero to 0.278 (mean 0.075). No pair of loci showed significant LD in two or more populations (*P* < 0.05), suggesting the independent segregation of the marker loci used. Twenty-eight loci deviated significantly from HWE (*P* < 0.01), including 12 gSSRs and 16 EST-SSRs (Supplementary Table S2). The 12 non-HWE gSSRs, along with gSSR loci (eight, including six non-HWE loci; Supplementary Fig. S1 and Table S2) that exhibited *F*_ST_ outlying values for the 16 populations, were excluded from subsequent population diversity and structure analyses.

High levels of putatively neutral (gSSR) diversity were revealed in *E. grandis* populations (Table 2), e.g. *H*_E_ ranging from 0.706 to 0.809 (mean 0.774) and *A*_R_ from 4.295 to 5.300 (mean 4.929). Specifically, the five northern populations (codes Pic, Cop, MS, Fin and Cre, mean *H*_O_ = 0.711) were significantly less diverse as compared to the remaining (southern) populations (mean *H*_O_ = 0.758, *P* = 0.007). The fixation index (*F*) per population suggested little evidence of inbreeding, with a range between zero and 0.127 (mean 0.037). The overall *F*_ST_ values were generally low as demonstrated by between-population comparisons (mean *F*_ST_ = 0.037; Supplementary Table S3) and AMOVA analysis (among-population variation percentage being 3.7%, *P* < 0.001; Supplementary Table S4), indicating a weak population structure.

**Figure 2:**
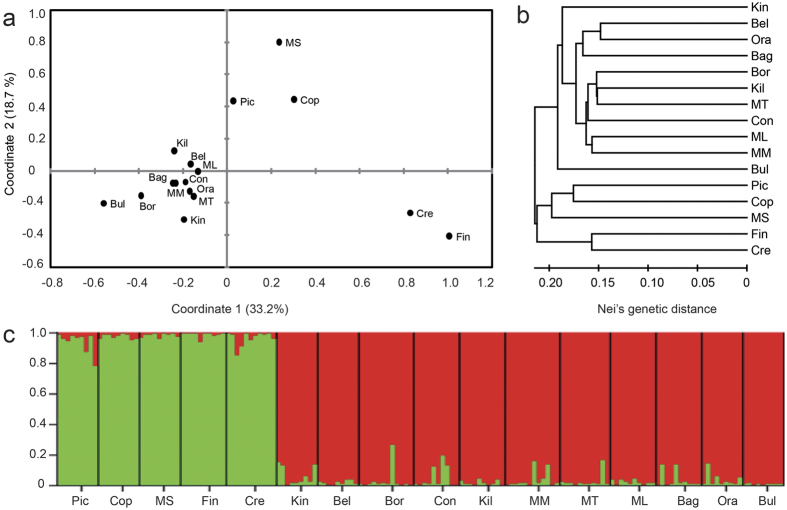
Genetic structure of 16 *Eucalyptus grandis* populations based on 31 putatively neutral gSSR loci. Full description of the populations can be found in Table 1. (**a**) Principal coordinates analysis (PCoA). (**b**) Unweighted pair group method with arithmetic mean (UPGMA) dendrogram. (**c**) Individual proportion and population membership to each of the clusters inferred in STRUCTURE analysis (*K* = 2).

Similar patterns of neutral population structure were observed in PCoA analysis, UPGMA dendrogram and STRUCTURE analysis (Fig. 2). For PCoA analysis (Fig. 2a), the first coordinate accounted for 33.2% of the variation and separated clearly the northern and southern populations. Further, the UPGMA dendrogram (Fig. 2b) based on Nei’s genetic distance confirmed the clustering of northern vs. southern populations, coinciding well with geographic distribution. Furthermore, the STRUCTURE analysis (Fig. 2c) illustrated the differentiation between the northern and southern populations, providing evidence for the presence of two genetically distinct clusters (i.e. *K* = 2).

### Climatic data and partitioning of populations

Of the 19 climatic variables obtained (http://www.worldclim.org/), only three were not correlated with each other, namely, mean annual temperature, isothermality and annual precipitation. The *k*-means analysis partitioned optimally the 16 populations into six, four and four groups in mean annual temperature, isothermality and annual precipitation, respectively (Table 1). Each group comprised two or more populations, with the only exception of one population in group 4 for isothermality partition.

### *F*_ST_ outlier loci

A total of 58 *F*_ST_ outlier loci were identified collectively using LOSITAN, ARLEQUIN and BAYESCAN against the three non-correlated climatic factors (Table 3), including 35 (31.8%, 24 positive and 11 balancing selections; Table 3), 22 (20.0%, 10 positive and 12 balancing selections; Table 3) and 20 (18.2%, 13 positive and seven balancing selections; Table 3) outliers for mean annual temperature, isothermality and annual precipitation, respectively. Four outliers were detected in all three climatic partitions, namely, EUCeSSR0755, EUCeSSR1070, Embra394 and EUCeSSR0893. In addition, the rates of EST-SSR outliers (20.0–33.8%) for all the climatic partitions were significantly greater (*P* = 0.006) than those of genomic SSRs (15.6–28.9%; Table 3).

LOSITAN, ARLEQUIN and BAYESCAN detected consistently the largest, medium and least numbers, respectively, of *F*_ST_ outlying loci for all the three climatic partitions (Table 3 and Supplementary Fig. S2). There were 11 (10.0%), 10 (9.1%) and five (4.5%, Table 3) outliers identified simultaneously in LOSITAN and ARLEQUIN analyses for mean annual temperature, isothermality and annual precipitation, respectively, while only one outlier locus EUCeSSR0893 (0.9%; Table 3) identified in both LOSITAN and ARLEQUIN was detected with BAYESCAN.

### Loci and alleles associated with climatic variables

**Figure 3:**
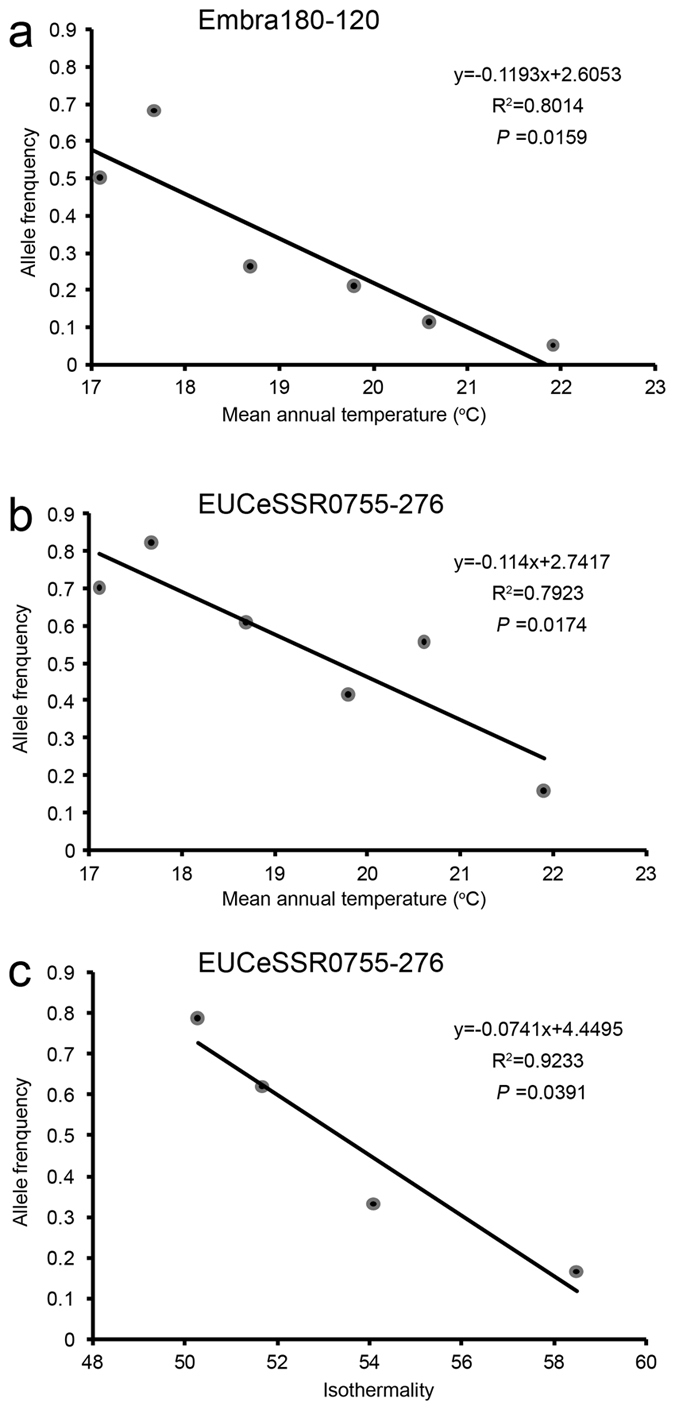
Linear regression for three significant associations between *F*_ST_ outlier allele frequencies and climatic variables. Each dot represents a group of homogeneous populations in *K*-means climatic partition. (**a**) The 120 bp allele of locus Embra180 associated with mean annual temperature. (**b**,**c**) The 276 bp allele of EUCeSSR0755 associated with mean annual temperature and isothermality, respectively.

A total of nine significant associations were detected between *F*_ST_ outlier allele frequencies and climatic variables, involving seven alleles from five loci (Table 4). Regression analysis indicated that three of the associations were significantly negatively linear (Fig. 3) while the remaining six were non-linear (Supplementary Fig. S3). Five, two and two alleles were significantly associated with mean annual temperature, isothermality and annual precipitation, respectively. All alleles exhibited significant association with only one climatic factor, with the exceptional allele EUCeSSR0755-276 bp associated with all the three climatic factors. In addition, two alleles (266 and 276 bp) of the same locus EUCeSSR0755 were significantly associated with the same climatic factor, mean annual temperature.

## Discussion

### High genetic diversity and low population structure in *E. grandis*

Despite the large geographical (latitudinal) range, *E. grandis* populations show high levels of neutral genetic diversity (mean *H*_E_ = 0.774; Table 2), consistent with SSR-derived population diversity in other eucalypt species, e.g. *H*_E_ being 0.82 in *E. globulus* Labill.^50^, 0.75 in *E. gomphocephala*^24^ and 0.739 in *E. urophylla* S.T. Blake^51^. Northern populations tend to be significantly less diverse than the southern populations, similar to the observations based on chloroplast DNA sequences^52^. It is possible that the relatively isolated northern populations have been colonized from the south^52^ as genetic diversity is generally higher in source populations than in younger populations derived from them^51^. Moreover, as *E. grandis* may cross with co-occurring close relatives such as *E. saligna* Smith, *E. robusta* Smith and *E. resinifera* Smith^21^, gene flow from the related species may have contributed to the high genetic diversity, in the southern populations in particular^52^. Additionally, all populations analysed here were sampled from a field trial, in which selection might have taken place during earlier seed germination, seedling establishment and juvenile survival stages, and the neutral genetic diversity could be thus more or less affected. Nevertheless, the overall high levels of genetic diversity may reflect the maintenance of large population size in *E. grandis*.

The low levels of population differentiation (mean *F*_ST_ = 0.037) are less than the mean of widespread eucalypt species, e.g. mean *F*_ST_ of 0.062 and 0.055 at restriction fragment length polymorphism (RFLP) and SSR loci, respectively^53^. These results confirm also earlier findings of limited population structure in *E. grandis* based on isozyme markers^23^. Generally speaking, forest tree species including eucalypts have weak population structure^54^. Such weak population structure, in light of the large geographical disjunctions in the natural range of *E. grandis* (e.g. populations MS vs. Fin and Cre vs. Kin) that could contribute as a barrier to gene flow and induce genetic differentiation, may reflect recent emergence of the disjunctions from a more continuous distribution due to climate change^52^.

In this study, besides the regions within *E. grandis* distribution range sampled earlier by Jones *et al.*^52^, an additional isolated region (populations Fin and Cre) in central coastal Queensland was included, providing a more comprehensive picture of the population diversity and structure. These results can have important implications for conservation and utilization of the species. The high levels of population diversity and certain genetic differentiation among populations detected herein could imply the great potential of further genetic resource exploitation. The populations featured with high diversity and/or rich private alleles, particularly those under potential threat by predicted climate change, should be considered in future conservation programs. For the purpose of association mapping studies, the population subdivision information will be of practical importance.

### Detection of *F*_ST_ outlier loci

Similar to the *k*-means partition of populations according to climatic variables implemented in genome scans with black spruce^43^, the 16 populations of *E. grandis* analysed were partitioned into homogeneous groups to identify *F*_ST_ outliers related with genetic divergence under specific environmental factors. Such climatic partitions may offer better odds to identify genes potentially involved in adaptation to a specific climate factor^43^, and those outliers specific only to a climatic factor could provide evident support in this respect. Based on the climatic partition of population groups, 35, 22 and 20 outlier loci (Table 3) were identified collectively for mean annual temperature, isothemality and annual precipitation, respectively. Of these, eight, eight and five loci presented higher genetic differentiation (*F*_ST_) values in at least two outlier detection methods than expected in the respective climatic variables, suggesting that they could have been involved in divergent selection among homogeneous population groups. In contrast, 11, 12 and seven loci presented lower *F*_ST_ values than expected in the respective variables, signaling balancing selection within population groups and/or homogeneous selection among groups. In addition, quantitative trait differentiation coefficients (*Q*_ST_) are 0.33 and 0.28 in 7-year-old tree height and diameter at breast height, respectively, for a range-wide provenance/family trial of *E. grandis* (Trial E110-1^55^; J Luo, China Eucalypt Research Centre, China, pers. comm., assuming an overall coefficient of relatedness of 0.4 amongst open-pollinated siblings), being much larger than the mean *F*_ST_ (0.037; Supplementary Table S3) and thus indicating the role of divergent selection on quantitative traits^56^ (our field trial was not used for estimating *Q*_ST_ due to abnormal growth caused by infection of gall wasp, *Leptocybe invasa* Fisher and La Salle).

The rates of *F*_ST_ outlier loci detected totally with all the three methods (18.2–31.8% depending on climatic partition; Table 3) are parallel to those reported in several forest trees, e.g. 16.7% (3/18) at genomic SSR and EST-SSR markers in *E. gomphocephala*^24^, 22–53% at gene-linked loci in *Quercus robur* L. and *Q. petraea* (Matt.) Liebl.^57^ and 31.3% (10/32) at EST-SSRs in *Castanopsis fargesii* Franchet^58^. However, such rates are generally greater than those reported by other studies in plants and animals, e.g. 12.0% (3/25) at SSRs in eelgrass (*Zosteria marina* L.)^59^, 11.4 % (5/44) at genomic SSRs and EST-SSRs in *Q. robur* and *Q. ellipsoidalis* E.J. Hill^60^ and 0.3% (18/6145) at restriction site associated DNAs (RADs) in kokanee salmon (*Oncorhynchus nerka* Walbaum)^61^. Given that only one *F*_ST_ outlier (0.9%; Table 3) was detected with the more conservative Bayesian method of BAYESCAN, the high proportion of outliers reported here is likely attributable to the utilization of relaxed summary-statistics approaches conducted in LOSITAN and ARLEQUIN. As a consequence, false positive outliers may exist, which remain still a challenge to be separated from real outliers^59^. Moreover, besides some common outliers (5–11 depending on climatic partition; Table 3) between LOSITAN and ARLEQUIN analyses, higher proportions of outliers were consistently detected by LOSITAN for the three climatic partitions as compared with those of ARLEQUIN, but it is impossible to figure out which detection method is more powerful. Furthermore, given the multiplicity of natural habitats of *E. grandis* and other eucalypt species, the *F*_ST_ outliers identified herein can be used as candidates for further genome scans against extended population samples.

### Significant loci associated with climatic variables

Five (8.6%) of the *F*_ST_ outlier loci showed significant associations between allelic frequencies and climatic variations (Table 4). However, the remaining *F*_ST_ outliers should not be considered as false positives as they may be involved in adaptation on only a fraction of the climatic gradient or in part of adaptation complexes resulting from epistatic interactions^43^. Of the five significant loci, two (EUCeSSR1044 and Embra394) were homologous to known gene or predicted protein, but three (Embra180, EUCeSSR0755 and EUCeSSR0849) did not have significant matches or known functions when they were BlastX searched against the NCBI non-redundant protein database (http://blast.ncbi.nlm.nih.gov/Blast.cgi).

The locus EUCeSSR1044, associated significantly with annual precipitation, was functionally annotated as C3HC4 type RING finger protein. Similarly, two *C3HC4 RING finger* genes were identified to be associated with local precipitation in black spruce^43^. In eukaryotes, C3HC4 type RING finger proteins act as E3 ubiquitin ligases, targeting numerous intracellular regulators in a wide range of physiological processes^62^,^63^, including abscisic acid (ABA) mediated drought stress responses in plants^64^,^65^. The plant hormone ABA can induce stomatal closure to mitigate transpirational water loss and is the master modulator of drought responses^64^. Changes in *C3HC4 RING finger* gene expression have been reported in response to drought stress in many plants, such as *Arabidopsis thaliana* (L.) Heynh.^64^,^65^ and *Populus* species^66^−^68^. Thus, it is not surprising to find significant associations between C3HC4 type RING finger protein and annual precipitation as critically low precipitations can cause drought stress.

The marker Embra394, significantly associated with mean annual temperature, was functionally annotated as thionin-like protein 2. The thionin-like proteins have been well documented in plants to be involved in protection against pathogens, including bacteria and fungi^69^. Furthermore, in wheat (*Triticum aestivum* L.), thionin-like genes can express differently in response to heat stress^70^ or be involved in pathogen resistance induced by low temperatures^71^. As similarities in adaptation exist among phylogenetically remote plants^43^, thionin-like proteins may take part in temperature responses in *Eucalyptus*.

The remaining three loci were of unknown function, including EUCeSSR0755 which was associated with all three climatic variables and EUCeSSR0849 which was related to both mean annual temperature and isothermality (Table 4). These EST-derived loci may represent candidate genes for physiological response to environmental variation. Alternatively, they may be artefacts of genetic hitchhiking^11^, in which neutral markers are linked to a single gene under positive selection.

### Implications for adaptation genomics in perennial trees

The relatively large number of *F*_ST_ outlier loci (58 in total, 52.7%; Table 3) may indicate that adaptive genetic variation is a genome-wide phenomenon^72^, in which multiple loci of small adaptive effect are spread across the genome. Similarly, relatively large numbers of *F*_ST_ outliers were revealed by genome scan in some other broadleaved trees (e.g. *Po. alba* L.^73^) and conifers (e.g. black spruce^43^).

Divergent natural selection can change allele frequencies and thereby increase the adaptive trait value of a population and the number of individuals with the fitness traits, leading to local adaptation^6^,^7^. Here, seven alleles were revealed to be significantly associated with the three climatic variables in *E. grandis*, indicating the importance of these climate factors as selective agents. Most of the significant alleles were associated with mean annual temperature followed by isothermality and annual precipitation, suggesting the critical role of mean annual temperature in determining adaptive responses. Many studies have demonstrated the influence of these climatic agents on selection and adaptation in forest trees, such as black spruce^43^, *E. gomphocephala*^24^, *C. fargesii*^58^ and *Pi. albies* (L.) Karst.^74^. With the occurrence of globally rapid climatic change, in temperature in particular, these results could inform the adaptive responses of perennial trees to the environment. The existence of divergent natural selection, in addition to high genetic diversity, implies the great potential for evolution of tree populations in adaptive traits in the face of climate change^6^. The populations that harbour the highest frequency of favourable alleles underlying the adaptive traits will be the most adaptive, and *vice versa*, under a climatic scenario of pronounced warming temperature and declining rainfall^19^.

Only one or two alleles of each significantly associated locus showed population variation with climatic variables, which may indicate that the significant alleles alter protein function in a way that the remaining alleles do not^24^. Given that none of the five significant loci have been directly investigated for their functional roles in perennial trees, these loci and their variation patterns described here will provide a foundation on which further functional characterisation work can rely.

In conclusion, the widespread woody species *E. grandis* provides an unusual opportunity to study local population adaptation in perennial trees. Although a relatively low density of multi-allelic loci were used for genome scans (only 110 SSRs spanning the *E. grandis* genome), we found evident footprints of divergent selection at a suite of loci. Two loci that showed significant associations with climatic variables represent alleles of putative genes with known functional importance for response to climatic factors. In addition, high genetic diversity levels and weak population structure were detected in *E. grandis* natural populations. These results have implications for understanding the genomic basis of adaptation to climates in perennial woody trees as well as for conservation and utilization of the important hardwood tree *E. grandis*. Further genetic association and expression studies would be required to confirm the functional role of putative genes. Also, next-generation sequencing technologies, including those for genotyping of multi-allelic markers (e.g. short tandem repeats^75^), will help to investigate fine-scale genome-wide patterns of natural selection and local adaptation.

## Additional Information

**How to cite this article**: Song, Z. *et al.* Genome scans for divergent selection in natural populations of the widespread hardwood species *Eucalyptus grandis* (Myrtaceae) using microsatellites. *Sci. Rep.* **6**, 34941; doi: 10.1038/srep34941 (2016).

## Supplementary Material

Supplementary Information

## Figures and Tables

**Table 1:** *Eucalyptus grandis* populations, their origins and sample size as well as the mean values of three non-correlated climatic variables (during 1950–2000).

No.	Code	Population	Latitude (S)	Longitude (E)	Altitude (m)	N	MAT (°C)	MAT partition	IT	IT partition	AP (mm)	AP partition
1	Pic	Piccaninny CK Windsor, QLD	16°13′	144°58′	1,160	9	19.7	5	60.1	2	1,352	3
2	Cop	Copperlode, QLD	16°58′	145°40′	425	9	21.7	4	56.9	2	2,195	2
3	MS	MT Spec SF Paluma, QLD	18°56′	146°07′	850	9	20.4	3	54.1	4	1,001	4
4	Fin	Finch Hatton Gorge, QLD	21°04′	148°37′	200	10	22.1	4	51.1	1	1,336	3
5	Cre	Credition SF, QLD	21°13′	148°28′	720	11	19.8	5	51.6	1	983	4
6	Kin	Kin Kin, QLD	26°12′	153°10′	40	9	20.8	3	50.6	3	1,517	1
7	Bel	Belli, QLD	26°29′	152°50′	100	9	19.9	5	51.7	1	1,498	1
8	Bor	Borumba Range, QLD	26°35′	152°36′	500	11	18.4	1	51.7	1	1,319	3
9	Con	Connondale, QLD	26°40′	152°36′	560	10	17.5	2	51.5	1	1,384	3
10	Kil	Kilcop Creek, QLD	26°45′	152°35′	400	10	18.0	2	51.8	1	1,276	3
11	MM	MT Mee, QLD	27°08′	152°43′	200	12	19.0	1	51.9	1	1,178	3
12	MT	MT Tamborine, QLD	27°55′	153°11′	500	12	17.7	2	52.0	1	1,522	1
13	ML	MT Lindsay, QLD	28°21′	152°45′	340	10	17.2	6	51.8	1	1,262	3
14	Bag	Bagawa, NSW	30°07′	152°54′	440	10	17.0	6	50.0	3	1,841	2
15	Ora	Orara West SF, NSW	30°20′	153°00′	293	9	17.5	2	50.3	3	1,951	2
16	Bul	Bulahdelah SF, NSW	32°20′	152°15′	20	9	17.7	2	50.2	3	1,307	3
Total						159		6		4		4

Using a *k*-means analysis^42^ on each of the non-correlated climatic variables, climatic partitioning assigned the populations to four or six groups. QLD, Queensland; MT, mountain; SF, state forest; NSW, New South Wales; *N*, number of individuals; MAT, mean annual temperature; IT, isothermality; AP, annual precipitation.

**Table 2:** Genetic diversity parameters of *E. grandis* populations based on 31 putatively neutral gSSR loci.

No.	Pop. code^†^	*A*_NA_	*N*_PA_	*H*_O_	*H*_E_	*F*	*A*_R_
1	Pic	8.484	4	0.708	0.753	0.049	4.624
2	Cop	8.161	8	0.701	0.716	0.009	4.295
3	MS	7.903	8	0.717	0.748	0.040	4.557
4	Fin	8.903	7	0.692	0.706	0.005	4.459
5	Cre	9.903	4	0.738	0.736	–0.002^‡^	4.693
6	Kin	8.613	7	0.761	0.801	0.050	5.280
7	Bel	8.323	3	0.760	0.791	0.045	5.256
8	Bor	10.548	6	0.752	0.801	0.055	5.235
9	Con	8.484	6	0.769	0.803	0.039	5.199
10	Kil	9.032	6	0.752	0.773	0.029	5.000
11	MM	11.516	10	0.709	0.809	0.127	5.144
12	MT	11.355	8	0.769	0.806	0.056	5.077
13	ML	9.065	8	0.768	0.775	0.004	4.918
14	Bag	9.516	13	0.742	0.792	0.065	4.953
15	Ora	8.290	11	0.788	0.804	0.022	5.300
16	Bul	8.258	12	0.767	0.770	0.000	4.870
Mean (SE)		9.147 (1.129)	7.560 (2.851)	0.743 (0.009)	0.774 (0.005)	0.037 (0.011)	4.929 (0.318)

^†^See Table 1 for full description of the populations. ^‡^The negative *F* value was treated as zero. *A*_NA_, average number of alleles per locus; *N*_PA_, number of private alleles; *H*_O_, observed heterozygosity; *H*_E_, expected heterozygosity; *F*, fixation index; *A*_R_, allelic richness.

**Table 3:** *F*_ST_ outliers detected for the three un-correlated climatic factors in LOSITAN^45^, ARLEQUIN^47^ and BAYESCAN^49^.

SSR locus	MAT			Isothermality			Annual precipitation		
	L^†^	A^‡^	B^§^	L^†^	A^‡^	B^§^	L^†^	A^‡^	B^§^
EUCeSSR1061		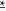							
Embra180	**			*	*				
EUCeSSR347	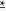	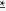							
Embra98				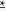					
EUCeSSR0502				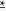	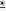				
EUCeSSR0224		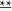							
EUCeSSR0276								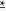	
EUCeSSR0979							**		
Embra227		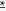							
Embra280	**						**		
EUCeSSR313		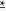							
EUCeSSR384	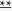	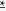		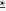					
EUCeSSR0599		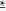							
Embra130							**		
EUCeSSR0035							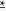		
EUCeSSR151				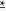				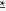	
EUCeSSR686	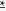	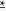							
Embra242				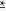			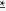		
Embra120	*								
EUCeSSR626								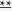	
EUCeSSR0455							**	*	
Embra358	*								
Embra304	***								
EUCeSSR0103	*								
Embra188	**	*							
EUCeSSR0906	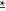			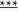					
Embra37				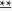					
Embra187	**								
EUCeSSR0755	**	**			*		***	**	
Embra233	*								
EUCeSSR231					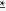				
EUCeSSR0620				*					
EUCeSSR880	*								
EUCeSSR479				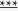					
EUCeSSR1042	*	**					**	*	
Embra83				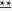					
Embra369	*								
EUCeSSR1087				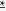	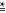				
EUCgSSR21				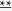					
EUCeSSR522	*								
Embra197								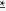	
EUCeSSR1070	***			**	*		*		
Embra88	**	**		*	*				
Embra53	*			*	*				
EUCeSSR0497	*								
EUCeSSR0845		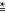							
Embra217							*		
EUCeSSR0679	*								
EUCeSSR1044							*		
EUCeSSR0568								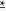	
Embra394	***	***		***	**		***	**	
EUCeSSR0893	***	**	***	***	**	**	***	**	***
Embra269							*		
EUCeSSR292	***	**		*	**				
EUCeSSR349							*		
EUCeSSR209	*								
EUCeSSR0849	**	*		*	*				
EUCeSSR1145		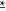							
Sub-total	28 (24 + 4)	18 (8 + 10)	1	20 (9 + 11)	12 (9 + 3)	1	15 (13 + 2)	10 (5 + 5)	1
L ∩ A	11 (10.0%; 8 + 3)^¶^			10 (9.1%; 8 + 2)^¶^			5 (4.5%; 5 + 0)^¶^		
L ∩ A ∩ B	1 (0.9%)			1 (0.9%)			1 (0.9%)		
L ∪ A ∪ B	35 (31.8%; 24 + 11)^¶^			22 (20.0%; 10 + 12)^¶^			20 (18.2%; 13 + 7)^¶^		
gSSRs	13 (28.9%)^††^			8 (17.8%)^††^			7 (15.6%)^††^		
EST-SSRs	22 (33.8%)^††^			14 (21.5%)^††^			13 (20.0%)^††^		
Total	58 (52.7%; 32 + 26)^¶^								

^†^Confidence levels of the distribution relative to neutral selection: *, above the 95% level; **, above the 99% level; ***, above the 99.9% level; 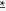, below the 5% level; 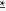

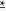
, below the 1% level; 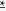

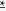

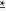
, below the 0.1% level. ^‡^Significance levels at the 95% confidence: *, 0.01 < *P* ≤ 0.05; **, 0.001 < *P* ≤ 0.01; ***, *P* ≤ 0.001. Significance levels at the 5% confidence: 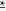, 0.01 < *P* ≤ 0.05; 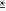

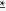
, 0.001 < *P* ≤ 0.01. ^§^Grades of evidence of selection: **, very strong evidence; ***, decisive evidence. ^¶^Percentage of the total number of markers used (110). ^††^Percentage of the total number of genomic SSR markers (45) or EST-SSR markers (65) used. The underlined asterisks and numbers are for balancing selection loci. MAT, mean annual temperature; L, LOSITAN; A, ARLEQUIN; B, BAYESCAN; L ∩ A, LOSITAN and ARLEQUIN; L ∩ A ∩ B, LOSITAN, ARLEQUIN and BAYESCAN; L ∪ A ∪ B, LOSITAN, ARLEQUIN or BAYESCAN.

**Table 4:** Loci and alleles significantly associated with the three non-correlated climatic variables in *E. grandis*.

Locus-allele (bp)	Scaffold^†^	MAT	Isothermality	MAP	Putative function at E ≤ 10^−5^ (Organism; BlastX E value)
Embra180-120	1	+			No significant match
EUCeSSR0755-266	6	+			No significant match
EUCeSSR0755-276	6	+	+	+	
EUCeSSR1044-406	10			+	Zinc finger, C3HC4 type (RING finger) protein (*Medicago truncatula*; 5E-69)
Embra394-215	10	+			Predicted: thionin-like protein 2 (*Eucalyptus grandis*; 7E-34)
EUCeSSR0849-212	11		+		Predicted: uncharacterized protein LOC104426570 (*Eucalyptus grandis*; 2E-26)
EUCeSSR0849-232	11	+			

^†^Scaffolds as aligned to *E. grandis* genome sequence (version 1.1, http://www.phytozome.net/eucalyptus.php). A plus sign (+) indicates the significant association detected by both G and Wald tests. MAT, mean annual temperature; MAP, mean annual precipitation.
