# Supplementary material for: Genome scans for divergent selection in natural populations of the widespread hardwood species Eucalyptus grandis (Myrtaceae) using microsatellites
Source: Sci Rep. 2016 Oct 17;6:34941. doi: 10.1038/srep34941 (PMC5066178; doi:10.1038/srep34941)
Supplement: Supplementary Information [file srep34941-s1.pdf]

Supplementary Information

Genome scans for divergent selection in natural populations of the widespread hardwood species *Eucalyptus grandis* (Myrtaceae) using microsatellites

Zhijiao Song[1,2,5†], Miaomiao Zhang[2,4,†], Fagen Li[2], Qijie Weng[2], Chanpin Zhou[2], Mei Li[2], Jie Li[2], Huanhua Huang[3], Xiaoyong Mo[4,*] & Siming Gan[1,2,*]

[1]State Key Laboratory of Tree Genetics and Breeding, Chinese Academy of Forestry, Xiangshan Road, Beijing 100091, China. [2]SFA Key Laboratory of Tropical Forestry, Research Institute of Tropical Forestry, Chinese Academy of Forestry, Longdong, Guangzhou 510520, China. [3]Guangdong Academy of Forestry, Longdong, Guangzhou 510520, China. [4]College of Forestry, South China Agricultural University, 284 Block, Wushan Street, Guangzhou 510642, China. [5]Baoshan University, Yuanzheng Road, Baoshan 678000, China. [†]These authors contributed equally to this work. *Correspondence and requests for materials should be addressed to X.M. (email: motree@163.com) or S.G. (email: siming.gan@ritf.ac.cn)

Supplementary Figures S1−S3 and Tables S1−S4

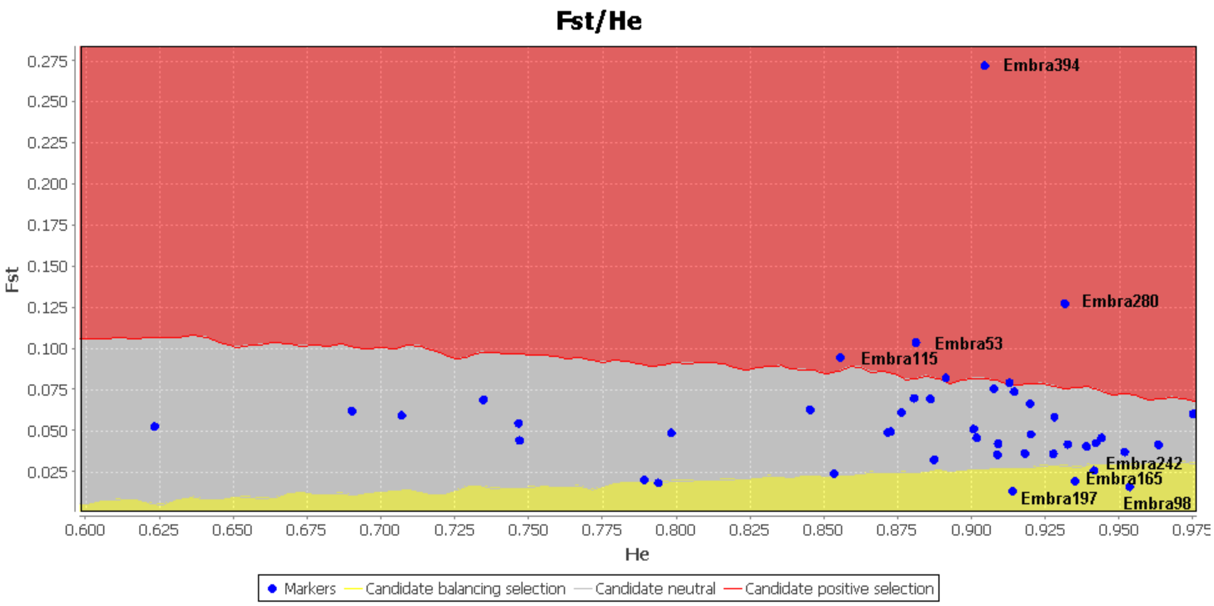

Supplementary Figure S1. Distribution of F_{ST} values for 45 gSSR loci at 95 % and 5 % confidence levels as detected with LOSITAN over the 16 *E. grandis* populations. The eight loci outside the confidence level of 95 % (four loci: Embra115, Embra280, Embra53 and Embra394) or 5 % (four loci: Embra98, Embra242, Embra197 and Embra165) were excluded from population diversity and structure analyses.

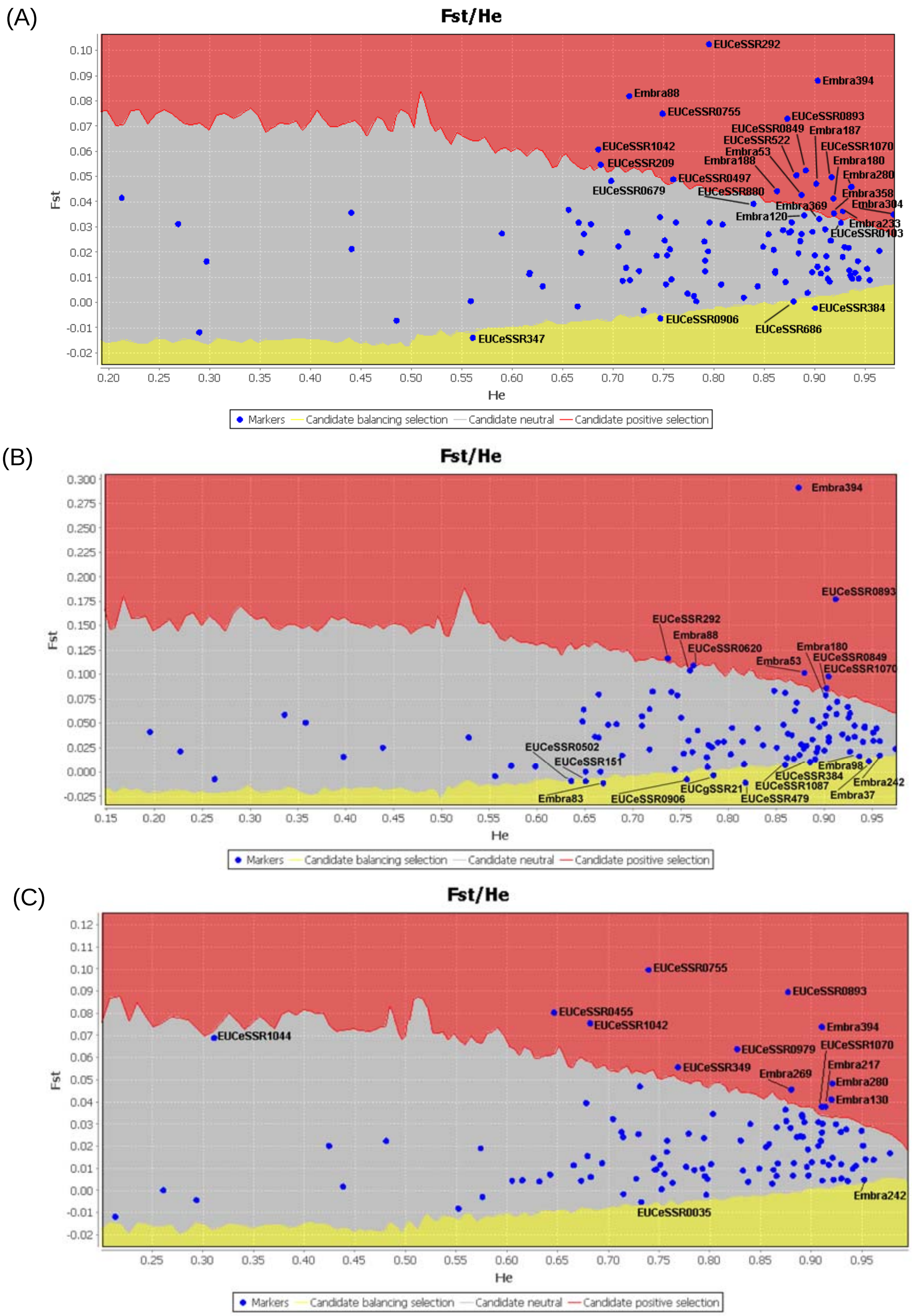

Supplementary Figure S2. Distribution of F_{ST} values for all the 110 SSR loci at 95 % and 5 % confidence levels as detected with LOSITAN over the three climatic partitions of *E. grandis* populations. (A) mean annual temperature; (B) isothermality; (C) annual precipitation.

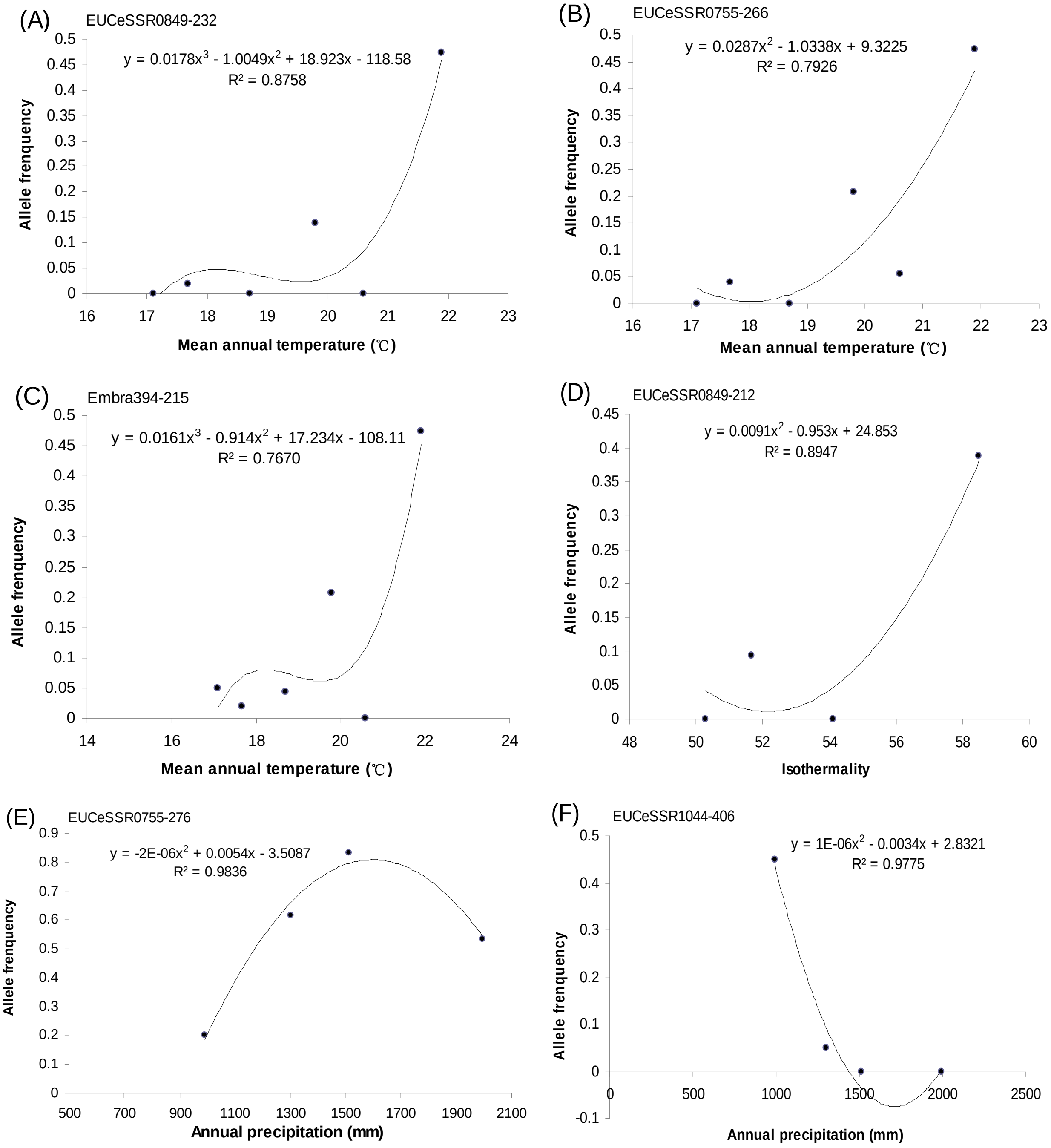

Supplementary Figure S3. Non-linear regression for six significant associations between F_{ST} outlier allele frequencies and climatic variables. Each dot represents a group of homogeneous populations in *K*-means climatic partition. (A, B, C) the alleles 232, 266 and 215 bp of loci EUCeSSR0849, EUCeSSR0755 and Embra394 associated significantly with mean annual temperature, respectively; (D) the 212 bp allele of EUCeSSR0849 associated significantly with isothermality; (E, F) the alleles 276 and 406 bp of loci EUCeSSR0755 and EUCeSSR1044 associated significantly with annual precipitation, respectively.

Supplementary Table S1. SSR markers, their functional annotations and physical positions in *E. grandis* genome sequence (version 1.1, http://www.phytozome.net/eucalyptus.php)

No.	SSR marker	Marker source		Putative function at $E \leq 10^{-5}$ [Organism; BlastX *E* value]	Physical position on *E. grandis* genome				
		GenBank accession	Reference		Scaffold	Start (bp)	End (bp)	Identity (%)	*E* value
1	EUCeSSR1061	Pr016588736	Zhou *et al.*, 2014	No significant match	1	8690373	8690158	100.0	1E-118
2	EUCeSSR1039	Pr016588725	Zhou *et al.*, 2014	VHS and GAT domain-containing protein [*Arabidopsis thaliana*; 2E-12]	1	10253398	10253454	98.3	1E-21
3	Embra180	BV682124	Brondani *et al.*, 2006	No significant match	1	16237395	16237490	95.8	8E-40
4	Embra366*	BV682836	Brondani *et al.*, 2006	Predicted: zinc-metallopeptidase, peroxisomal-like [*Eucalyptus grandis*; 5E-14]	1	28485149	28485018	97.0	6E-59
					5	20388443	20388312	97.0	6E-59
5	EUCeSSR485	Pr010837364	He *et al.*, 2012	Similar to hydroxylproline-rich glycoprotein family protein [*Vitis vinifera*; 2E-14]	1	28493543	28493043	99.4	0
6	Embra100	BV682837	Brondani *et al.*, 2006	Predicted: transparent testa 12-like [*Eucalyptus grandis*; 6E-19]	1	31237609	31237427	100.0	1E-98
7	EUCeSSR0333†	*NA*	Zhou *et al.*, 2014	No significant match	1	34568399	34568193	99.5	1E-110
8	EUCeSSR347	Pr010837326	He *et al.*, 2012	Protein exordium like 3 [*Arabidopsis thaliana*; 2E-09]	1	39170916	39171023	99.1	2E-51
9	EUCeSSR1136	Pr016588533	Zhou *et al.*, 2010	Light-harvesting complex I chlorophyll A/B-binding protein [*Medicago truncatula*; 9E-70]	2	6868216	6867926	99.3	6E-156
10	Embra98	BV682070	Brondani *et al.*, 2006	No significant match	2	12452178	12452441	89.4	8E-79
11	EUCeSSR0502	Pr016588591	Zhou *et al.*, 2014	No significant match	2	19852092	19851718	94.7	1E-166
12	EUCeSSR0224	Pr016588560	Zhou *et al.*, 2014	40S ribosomal protein s24 [*Cucumis melo* subsp. *melo*; 5E-78]	2	31569263	31569595	98.2	1E-171

No.	SSR marker	Marker source		Putative function at $E \leq 10^{-5}$ [Organism; BlastX E value]	Physical position on *E. grandis* genome				
		GenBank accession	Reference		Scaffold	Start (bp)	End (bp)	Identity (%)	*E* value
13	EUCeSSR0276	Pr016588569	Zhou *et al.*, 2014	DNA binding protein, putative [*Ricinus communis*; 2E-38]	2	37569214	37569335	98.4	8E-58
14	Embra333	BV682202	Brondani *et al.*, 2006	Transcriptional regulator SUPERMAN, putative [*Theobroma cacao*; 3E-17]	2	44098015	44098200	98.9	8E-96
15	Embra201	BV682141	Brondani *et al.*, 2006	No significant match	2	49421201	49421081	98.4	4E-57
16	EUCeSSR0930*	Pr016588682	Zhou *et al.*, 2014	Hypothetical VITISV_024902 [*Vitis vinifera*; 2E-25]	2	52914039	52913627	95.9	0
					5	17599471	17599056	97.6	0
17	EUCeSSR410	Pr010837342	He *et al.*, 2012	No significant match	2	60757654	60757309	99.4	0
18	EUCeSSR0979	Pr016588702	Zhou *et al.*, 2014	Hypothetical protein ARALYDRAFT_491646 [*Arabidopsis lyrata* subsp. *lyrata*; 9E-59]	3	7090509	7090657	100.0	1E-78
19	Embra115	BV682077	Brondani *et al.*, 2006	No significant match	3	11491555	11491340	98.6	1E-112
20	Embra227	BV682257	Brondani *et al.*, 2006	No significant match	3	13582421	13582257	97.6	4E-77
21	EUCeSSR1060	Pr016588522	Zhou *et al.*, 2010	Transmembrane protein, putative [*Medicago truncatula*; 5E-42]	3	13597010	13597399	96.4	0
22	Embra280	BV682184	Brondani *et al.*, 2006	No significant match	3	25904848	25904985	95.0	3E-52
23	Embra377	BV682218	Brondani *et al.*, 2006	No significant match	3	29789569	29789756	99.5	2E-97
24	Embra321	BV682198	Brondani *et al.*, 2006	Predicted: U-box domain-containing protein 35-like [*Eucalyptus grandis*; 1E-12]	3	39424661	39424785	99.2	9E-62
25	EUCeSSR313	Pr010837314	He *et al.*, 2012	No significant match	3	40581763	40582032	97.0	1E-135
26	EUCeSSR0857	Pr016588658	Zhou *et al.*, 2014	4-coumarate:CoA ligase [*Eucalyptus camaldulensis*; 1E-84]	3	42700560	42700039	94.9	0

No.	SSR marker	Marker source		Putative function at $E \leq 10^{-5}$ [Organism; BlastX *E* value]	Physical position on *E. grandis* genome				
		GenBank accession	Reference		Scaffold	Start (bp)	End (bp)	Identity (%)	*E* value
27	EUCeSSR384†	Pr010837336	He *et al*., 2012	Predicted: aspartate aminotransferase, chloroplastic-like [*Eucalyptus grandis*; 7E-22]	3	52236288	52236605	99.7	1E-176
28	Embra125	BV682083	Brondani *et al*., 2006	No significant match	3	52337372	52337243	99.2	4E-65
29	EUCeSSR0599	Pr016588608	Zhou *et al*., 2014	No significant match	4	7631897	7631979	98.8	6E-37
30	Embra130	BV682088	Brondani *et al*., 2006	No significant match	4	12276418	12276731	100.0	6E-66
31	EUCeSSR0035	Pr016588538	Zhou *et al*., 2014	GTP-binding protein rab-2 homolog [*Ciona intestinalis*; 1E-09]	4	17497132	17497192	92.2	3E-41
32	EUCeSSR0803	Pr016588646	Zhou *et al*., 2014	Adenosine 5' phosphosulfate reductase [*Populus tremula* x *P. alba*; 2E-58]	4	23911715	23911715	96.8	2E-71
33	EUCeSSR151	Pr010837266	He *et al*., 2012	No significant match	4	35595018	35594867	98.7	3E-75
34	EUCeSSR686†	Pr010837404	He *et al*., 2012	No significant match	4	36831665	36831351	99.4	1E-170
35	EUCeSSR0862	Pr016588660	Zhou *et al*., 2014	Arginine/serine-rich splicing factor 31 [*Arabidopsis lyrata* subsp. *lyrata*; 6E-13]	4	37785686	37785165	97.3	0
36	Embra242	BV682178	Brondani *et al*., 2006	No significant match	5	915509	915386	99.2	3E-61
37	EUCeSSR840	Pr010837435	He *et al*., 2012	Cinnamoyl-CoA reductase [*Ricinus communis*; 2E-69]	5	2602306	2602519	98.6	1E-108
38	Embra120	BV682081	Brondani *et al*., 2006	No significant match	5	3395332	3395550	96.8	2E-105
39	Embra41	BV682033	Brondani *et al*., 2006	No significant match	5	4524839	4525010	98.8	3E-75
40	EUCeSSR626	Pr010837388	He *et al*., 2012	Phosphoglycerate mutase, putative [*Ricinus communis*; 2E-44]	5	6125193	6124889	99.0	1E-163
41	Embra64	BV682234	Brondani *et al*., 2006	No significant match	5	12244555	12244784	94.8	1E-95
42	Embra111	BV682074	Brondani *et al*., 2006	No significant match	5	32787727	32787575	90.2	3E-50
43	Embra370	BV682215	Brondani *et al*., 2006	No significant match	5	41971188	41971422	89.4	1E-75

No.	SSR marker	Marker source		Putative function at $E \leq 10^{-5}$ [Organism; BlastX *E* value]	Physical position on *E. grandis* genome				
		GenBank accession	Reference		Scaffold	Start (bp)	End (bp)	Identity (%)	*E* value
44	EUCeSSR0455	Pr016588583	Zhou *et al.*, 2014	Hypothetical MTR_1g050940 [*Medicago truncatula*; 2E-15]	5	44544967	44545383	97.4	0
45	Embra358	BV682263	Brondani *et al.*, 2006	No significant match	5	47099301	47099219	100.0	3E-39
46	Embra304	BV682196	Brondani *et al.*, 2006	Hypothetical EUGRSUZ_E02918 [*Eucalyptus grandis*; 3E-23]	5	48126124	48126411	98.6	1E-149
47	EUCeSSR0103	Pr016588546	Zhou *et al.*, 2014	No significant match	5	54687233	54687339	95.4	9E-39
48	Embra188	BV682130	Brondani *et al.*, 2006	No significant match	5	58140803	58140472	99.1	3E-180
49	EUCeSSR0906	Pr016588672	Zhou *et al.*, 2014	Hypothetical MTR_2g104400 [*Medicago truncatula*; 3E-57]	5	60339030	60338720	98.4	1E-160
50	Embra37‡	*NA*	Brondani *et al.*, 2006	*NA*	5	68798617	68798743	–	–
51	EUCeSSR1134	Pr016588767	Zhou *et al.*, 2014	Hypothetical protein SORBIDRAFT_02g033820 [*Sorghum bicolor*; 6E-36]	5	73814771	73814707	100.0	1E-28
52	Embra187	BV682129	Brondani *et al.*, 2006	L-ascorbate oxidase [*Morus notabilis*; 4E-12]	6	7689616	7689783	96.4	2E-77
53	EUCeSSR346	Pr010837325	He *et al.*, 2012	GDP-L-galactose guanyltransferase [*Rosa roxburghii*; 1E-63]	6	9407858	9407463	98.2	0
54	EUCeSSR0755	Pr016588638	Zhou *et al.*, 2014	No significant match	6	14024833	14025371	94.5	0
55	EUCeSSR0705	Pr016588631	Zhou *et al.*, 2014	TCP domain class transcription factor [*Malus* x *domestica*; 1E-24]	6	15308410	15307977	99.1	0
56	Embra196	BV682136	Brondani *et al.*, 2006	No significant match	6	19255449	19255309	98.6	7E-69
57	Embra233	BV682169	Brondani *et al.*, 2006	No significant match	6	24277671	24277564	99.1	9E-52
58	EUCeSSR338	Pr010837321	He *et al.*, 2012	Dehydration-responsive element binding protein 3 [*Asparagus officinalis*; 2E-12]	6	34000528	34000073	93.5	0
59	EUCeSSR231	Pr010837287	He *et al.*, 2012	SLL1 protein [*Primula vulgaris*; 2E-28]	6	35460737	35461161	98.6	0

No.	SSR marker	Marker source		Putative function at $E \leq 10^{-5}$ [Organism; BlastX *E* value]	Physical position on *E. grandis* genome				
		GenBank accession	Reference		Scaffold	Start (bp)	End (bp)	Identity (%)	*E* value
60	EUCeSSR0959	Pr016588693	Zhou *et al*., 2014	F-actin capping protein beta subunit, putative [*Ricinus communis*; 1E-111]	6	36372826	36372684	93.7	3E-56
61	EUCeSSR739	Pr010837417	He *et al*., 2012	SNARE associated Golgi protein family [*Arabidopsis thaliana*; 2E-44]	6	39222673	39222816	98.6	3E-70
62	EUCeSSR0620	Pr016588613	Zhou *et al*., 2014	Cytochrome c-type biogenesis ccda-like chloroplastic protein 2-like [*Glycine max*; 4E-22]	6	42424251	42424207	100.0	9E-17
63	Embra135	BV682091	Brondani *et al*., 2006	No significant match	6	45979595	45979785	97.9	2E-92
64	Embra345	BV682206	Brondani *et al*., 2006	No significant match	6	46302395	46302603	96.7	3E-98
65	EUCeSSR0776	Pr016588642	Zhou *et al*., 2014	Low PSII accumulation 3 protein [*Arabidopsis thaliana*; 2E-48]	6	47938769	47938915	100.0	1E-77
66	EUCeSSR880	Pr010837440	He *et al*., 2012	Hypothetical protein [*Vitis vinifera*; 3E-47]	6	48283428	48283817	97.7	0
67	Embra81	BV682057	Brondani *et al*., 2006	Hypothetical EUGRSUZ_F043031 [*Eucalyptus grandis*; 2E-14]	6	53264142	53263801	99.4	0
68	EUCeSSR479	Pr010837362	He *et al*., 2012	Membrane-anchored endo-1,4-beta-glucanase [*Gossypium hirsutum*; 2E-12]	7	354399	354065	96.7	1E-165
69	EUCeSSR1042	Pr016588727	Zhou *et al*., 2014	Potassium channel 2 [*Populus tremula* x *P. tremuloides*; 6E-28]	7	9980448	9980798	97.2	1E-175
70	Embra7	BV682006	Brondani *et al*., 2006	No significant match	7	27204059	27203932	99.2	1E-63
71	Embra83	BV682059	Brondani *et al*., 2006	No significant match	7	47970364	47970228	87.6	2E-32
72	EUCeSSR0875	Pr016588663	Zhou *et al*., 2014	Major intrinsic protein PIPa2 [*Craterostigma plantagineum*;7E-58]	7	48997063	48996432	97.3	0
73	Embra369	BV682214	Brondani *et al*., 2006	No significant match	7	49475352	49475508	100.0	4E-83

No.	SSR marker	Marker source		Putative function at $E \leq 10^{-5}$ [Organism; BlastX *E* value]	Physical position on *E. grandis* genome				
		GenBank accession	Reference		Scaffold	Start (bp)	End (bp)	Identity (%)	*E* value
74	EUCeSSR1087	Pr016588747	Zhou *et al*., 2014	Leunig-like protein [*Arabidopsis thaliana*; 6E-64]	8	631971	631749	99.6	3E-120
75	EUCgSSR21	Pr010703614	He *et al*., 2011	Xyloglucan glycosyltransferase 6-like, putative [*Gossypium arboreum*; 1E-75]	8	9682075	9682227	100.0	5E-81
76	EUCeSSR683	Pr010837402	He *et al*., 2012	No significant match	8	11862587	11862800	99.1	4E-112
77	EUCeSSR522	Pr010837369	He *et al*., 2012	Esterase/lipase domain-containing protein [*Arabidopsis thaliana*; 1E-66]	8	15487717	15488064	100.0	0
78	Embra197	BV682137	Brondani *et al*., 2006	No significant match	8	33444065	33444325	99.6	1E-143
79	Embra150‡	*NA*	Brondani *et al*., 2006	*NA*	8	40661762	40661894	–	–
80	EUCeSSR0163	Pr016588551	Zhou *et al*., 2014	Actin depolymerizing factor 4 [*Gossypium hirsutum*; 7E-81]	8	44710190	44710369	98.9	3E-92
81	EUCeSSR1070	Pr016588742	Zhou *et al*., 2014	Predicted: CASP-like At3g23200 [*Vitis vinifera*; 1E-38]	8	45480459	45480283	96.6	6E-81
82	Embra88	BV682063	Brondani *et al*., 2006	No significant match	8	49475797	49476075	98.6	1E-145
83	Embra53	BV682042	Brondani *et al*., 2006	No significant match	8	56361679	56361842	100.0	1E-74
84	EUCeSSR0226	Pr016588561	Zhou *et al*., 2014	Nuclear transport factor, putative [*Ricinus communis*; 7E-30]	8	64788519	64788192	95.4	1E-149
85	EUCeSSR0497	Pr016588588	Zhou *et al*., 2014	ACT domain-containing protein, partial [*Silene latifolia*; 9E-70]	8	66205158	66204944	97.7	1E-105
86	EUCeSSR0845	Pr016588653	Zhou *et al*., 2014	NAC domain protein, IPR003441 [*Populus trichocarpa*; 5E-108]	9	961917	961845	100.0	2E-33
87	EUCeSSR1125	Pr016588762	Zhou *et al*., 2014	No significant match	9	3873376	3873504	97.7	1E-59

No.	SSR marker	Marker source		Putative function at $E \le 10^{-5}$ [Organism; BlastX *E* value]	Physical position on *E. grandis* genome				
		GenBank accession	Reference		Scaffold	Start (bp)	End (bp)	Identity (%)	*E* value
88	EUCeSSR0592	Pr016588607	Zhou *et al.*, 2014	Ubiquitin-like protein [*Arabidopsis thaliana*; 2E-32]	9	25296628	25296965	97.6	1E-170
89	EUCeSSR0909	Pr016588674	Zhou *et al.*, 2014	Hypothetical protein ARALYDRAFT_471189 [*Arabidopsis lyrata* subsp. *lyrata*; 6E-25]	9	27543468	27543222	93.1	5E-88
90	Embra217	BV682155	Brondani *et al.*, 2006	No significant match	9	38397371	38397559	100.0	1E-102
91	EUCeSSR0679	Pr016588623	Zhou *et al.*, 2014	No significant match	10	466663	466821	91.8	2E-62
92	EUCeSSR596	Pr010837383	He *et al.*, 2012	Nodulin MtN21 family protein [*Arabidopsis. lyrata* subsp. *lyrata*; 6E-41]	10	1657928	1658151	98.2	1E-100
93	Embra40	BV682229	Brondani *et al.*, 2006	No significant match	10	10043414	10043335	100.0	2E-37
94	EUCeSSR1044	Pr016588729	Zhou *et al.*, 2014	Zinc finger, C3HC4 type (RING finger) protein [*Medicago truncatula*; 5E-69]	10	22822844	22823183	99.1	0
95	EUCeSSR0568	Pr016588604	Zhou *et al.*, 2014	C-4 methyl sterol oxidase, putative [*Ricinus communis*; 3E-90]	10	26059346	26059495	99.3	3E-76
96	EUCeSSR0126	Pr016588548	Zhou *et al.*, 2014	High mobility group B3 protein [*Arabidopsis thaliana*; 2E-35]	10	31255535	31255866	99.1	7E-147
97	Embra394	BV682223	Brondani *et al.*, 2006	Predicted: thionin-like protein 2 [*Eucalyptus grandis*; 7E-34]	10	37285686	37285508	94.7	4E-80
98	Embra87	BV682062	Brondani *et al.*, 2006	No significant match	11	3914954	3914887	100.0	6E-30
99	Embra165	BV682112	Brondani *et al.*, 2006	No significant match	11	4343680	4343529	100.0	2E-80
100	Embra326	BV682200	Brondani *et al.*, 2006	No significant match	11	6199457	6199533	98.7	6E-34
101	EUCeSSR1117	Pr016588761	Zhou *et al.*, 2014	No significant match	11	9172710	9172816	99.1	8E-51
102	EUCeSSR0893	Pr016588666	Zhou *et al.*, 2014	No significant match	11	18351676	18351451	91.6	2E-69
103	Embra269	BV682181	Brondani *et al.*, 2006	No significant match	11	20369552	20369725	98.3	2E-87

No.	SSR marker	Marker source		Putative function at $E \leq 10^{-5}$ [Organism; BlastX *E* value]	Physical position on *E. grandis* genome				
		GenBank accession	Reference		Scaffold	Start (bp)	End (bp)	Identity (%)	*E* value
104	EUCeSSR1021	Pr016588718	Zhou *et al*., 2014	Succinic semialdehyde reductase isofom1 [*Solanum lycopersicum*; 5E-121]	11	30521569	30521634	95.5	3E-22
105	EUCeSSR292	Pr010837306	He *et al*., 2012	LAX4 protein [*Solanum lycopersicum*; 5E-10]	11	38058752	38058500	97.2	1E-123
106	EUCeSSR349	Pr010837328	He *et al*., 2012	Histone h2 [*Populus trichocarpa*; 7E-21]	11	40253366	40253636	99.3	1E-146
107	EUCeSSR209	Pr010837283	He *et al*., 2012	60S ribosomal protein L12 [*Capsicum annuum*; 3E-104]	11	41279073	41278307	98.2	0
108	EUCeSSR0849	Pr016588654	Zhou *et al*., 2014	Predicted: uncharacterized protein LOC104426570 (*Eucalyptus grandis*; 2E-26)	11	41365756	41365569	99.5	6E-99
109	Embra258	BV682180	Brondani *et al*., 2006	No significant match	11	45979595	45979785	97.9	1E-92
110	EUCeSSR1145	Pr016588769	Zhou *et al*., 2014	Heavy-metal-associated domain-containing protein [*Arabidopsis thaliana*; 3E-32]	14	855263	855161	98.1	2E-46

* Each of Embra366 and EUCeSSR0930 had two matches on different scaffolds in *E. grandis* genome sequence.

† EUCeSSR0333, EUCeSSR384 and EUCeSSR686 were homologous to EST3 (Acuña *et al*., 2012), Embra243 (also Embra286, Brondani *et al*., 2006) and Embra1481 (Faria *et al*., 2011), respectively.

‡ No marker sequence was available for Embra37 and Embra150, and their primer sequences were thus used to align with *E. grandis* genome.

Supplementary Table S2. Genetic parameters for 110 polymorphic SSR loci in *E. grandis* populations

No.[*]	SSR locus	N_A[†]	H_O	H_E	F	F_{IT}	F_{IS}	F_{ST}	PIC[‡]	NAF	A_R	HWE[§]
1	EUCeSSR1061	15	0.641	0.732	0.115	0.175	0.172	0.004	0.782	0.066	2.945	**
2	EUCeSSR1039	6	0.592	0.610	0.025	0.103	0.103	0.000	0.703	0.041	2.459	**
3	Embra180	20	0.724	0.795	0.099	0.194	0.137	0.066	0.898	0.047	3.479	
4	Embra366	25	0.771	0.784	0.010	0.121	0.080	0.044	0.870	0.027	3.336	
5	EUCeSSR485	8	0.694	0.701	0.007	0.067	0.058	0.009	0.710	0.016	2.768	
6	Embra100	21	0.449	0.778	0.440	0.500	0.481	0.036	0.861	0.184	3.490	**
7	EUCeSSR0333	17	0.556	0.785	0.294	0.366	0.341	0.038	0.873	0.127	3.351	
8	EUCeSSR347	5	0.787	0.545	-0.459	-0.387	-0.412	0.018	0.519	0.001	2.168	
9	EUCeSSR1136	29	0.435	0.767	0.431	0.495	0.502	-0.014	0.824	0.177	3.240	
10	Embra98[¶]	24	0.688	0.862	0.203	0.262	0.252	0.014	0.930	0.091	3.625	**
11	EUCeSSR0502	9	0.758	0.557	-0.367	-0.268	-0.304	0.027	0.644	0.004	2.274	**
12	EUCeSSR0224	12	0.979	0.738	-0.332	-0.256	-0.279	0.018	0.768	0.000	2.904	**
13	EUCeSSR0276	19	0.467	0.669	0.296	0.385	0.366	0.031	0.766	0.127	2.874	
14	Embra333	21	0.811	0.848	0.043	0.130	0.097	0.037	0.923	0.035	3.606	
15	Embra201	31	0.652	0.874	0.256	0.314	0.297	0.024	0.958	0.123	3.772	
16	EUCeSSR0930	5	0.303	0.275	-0.081	-0.044	-0.044	0.001	0.259	0.014	1.561	
17	EUCeSSR410	14	0.615	0.778	0.225	0.285	0.279	0.008	0.837	0.099	3.299	
18	EUCeSSR0979	12	0.613	0.667	0.079	0.215	0.134	0.093	0.788	0.033	2.985	
19	Embra115[¶]	18	0.326	0.733	0.560	0.614	0.587	0.064	0.850	0.222	3.235	**
20	Embra227	20	0.784	0.809	0.026	0.131	0.109	0.025	0.886	0.037	3.383	
21	EUCeSSR1060	9	0.586	0.647	0.079	0.164	0.157	0.008	0.665	0.078	2.642	
22	Embra280[¶]	25	0.264	0.737	0.659	0.751	0.736	0.057	0.816	0.278	3.533	**
23	Embra377	23	0.448	0.808	0.450	0.508	0.499	0.017	0.871	0.188	3.575	**
24	Embra321	8	0.532	0.560	0.026	0.159	0.116	0.048	0.576	0.039	2.322	
25	EUCeSSR313	15	0.643	0.665	0.025	0.125	0.111	0.015	0.754	0.032	2.766	
26	EUCeSSR0857	15	0.789	0.829	0.046	0.123	0.108	0.017	0.882	0.000	3.409	
27	EUCeSSR384	20	0.929	0.839	-0.110	-0.034	-0.054	0.019	0.890	0.005	3.442	

No.[*]	SSR locus	N_A[†]	H_O	H_E	F	F_{IT}	F_{IS}	F_{ST}	PIC[‡]	NAF	A_R	HWE[§]
28	Embra125	28	0.855	0.854	-0.002	0.097	0.056	0.043	0.937	0.023	3.662	
29	EUCeSSR0599	15	0.756	0.747	-0.039	0.117	0.094	0.026	0.819	0.049	3.189	
30	Embra130	23	0.720	0.823	0.122	0.214	0.189	0.031	0.913	0.067	3.539	
31	EUCeSSR0035	8	0.582	0.679	0.132	0.247	0.248	-0.002	0.762	0.077	2.789	
32	EUCeSSR0803	5	0.226	0.540	0.582	0.604	0.605	-0.002	0.588	0.201	2.248	
33	EUCeSSR151	4	0.660	0.584	-0.125	-0.054	-0.072	0.017	0.604	0.020	2.286	**
34	EUCeSSR686	14	0.911	0.829	-0.101	-0.028	-0.044	0.015	0.874	0.009	3.378	
35	EUCeSSR0862	17	0.810	0.696	-0.174	-0.073	-0.109	0.032	0.765	0.014	2.884	
36	Embra242[¶]	34	0.793	0.890	0.110	0.171	0.165	0.006	0.947	0.062	3.721	**
37	EUCeSSR840	8	0.695	0.706	0.016	0.131	0.086	0.048	0.784	0.032	2.924	**
38	Embra120	20	0.773	0.816	0.053	0.141	0.105	0.039	0.886	0.021	3.438	
39	Embra41	20	0.770	0.826	0.070	0.152	0.122	0.034	0.900	0.034	3.488	
40	EUCeSSR626	9	0.974	0.647	-0.527	-0.436	-0.458	0.015	0.642	0.000	2.513	
41	Embra64	18	0.715	0.675	-0.064	0.035	-0.008	0.042	0.745	0.008	2.838	
42	Embra111	31	0.693	0.853	0.188	0.269	0.247	0.030	0.937	0.084	3.656	
43	Embra370	16	0.501	0.719	0.312	0.369	0.347	0.033	0.779	0.116	2.981	
44	EUCeSSR0455	8	0.541	0.580	0.065	0.172	0.122	0.058	0.615	0.042	2.447	**
45	Embra358	22	0.761	0.846	0.101	0.178	0.156	0.027	0.922	0.045	3.577	
46	Embra304	52	0.355	0.865	0.590	0.632	0.623	0.024	0.965	0.267	3.835	**
47	EUCeSSR0103	25	0.633	0.818	0.227	0.327	0.297	0.042	0.916	0.126	3.562	
48	Embra188	26	0.460	0.721	0.357	0.458	0.433	0.045	0.828	0.141	3.262	
49	EUCeSSR0906	11	0.833	0.713	-0.176	-0.115	-0.118	0.003	0.725	0.001	2.839	
50	Embra37	32	0.820	0.870	0.056	0.119	0.101	0.020	0.934	0.038	3.654	
51	EUCeSSR1134	17	0.752	0.792	0.054	0.132	0.105	0.030	0.851	0.037	3.265	**
52	Embra187	31	0.575	0.774	0.255	0.358	0.314	0.066	0.884	0.087	3.397	**
53	EUCeSSR346	25	0.625	0.835	0.253	0.309	0.296	0.020	0.907	0.119	3.512	
54	EUCeSSR0755	9	0.514	0.582	0.119	0.304	0.179	0.151	0.680	0.057	2.689	
55	EUCeSSR0705	3	0.209	0.200	-0.019	0.098	0.042	0.060	0.316	0.021	1.434	
56	Embra196	26	0.465	0.613	0.229	0.312	0.282	0.041	0.688	0.091	2.663	

No.[*]	SSR locus	N_A[†]	H_O	H_E	F	F_{IT}	F_{IS}	F_{ST}	PIC[‡]	NAF	A_R	HWE[§]
57	Embra233	20	0.780	0.794	0.026	0.156	0.106	0.056	0.863	0.030	3.511	
58	EUCeSSR338	12	0.369	0.665	0.449	0.503	0.496	0.014	0.696	0.182	2.710	
59	EUCeSSR231	5	0.623	0.446	-0.379	-0.326	-0.359	0.025	0.444	0.000	1.961	
60	EUCeSSR0959	6	0.263	0.250	-0.059	0.021	-0.006	0.026	0.271	0.022	1.566	**
61	EUCeSSR739	7	0.633	0.662	0.049	0.113	0.086	0.028	0.680	0.046	2.623	
62	EUCeSSR0620	9	0.798	0.633	-0.260	-0.163	-0.218	0.044	0.644	0.003	2.563	
63	Embra135	21	0.793	0.778	-0.023	0.101	0.049	0.055	0.870	0.024	3.325	
64	Embra345	22	0.913	0.790	-0.160	-0.069	-0.100	0.029	0.833	0.001	3.224	
65	EUCeSSR0776	3	0.404	0.366	-0.106	0.021	-0.049	0.067	0.383	0.014	1.860	
66	EUCeSSR880	26	0.611	0.757	0.191	0.268	0.247	0.029	0.832	0.095	3.144	
67	Embra81	22	0.837	0.786	-0.073	0.038	-0.013	0.051	0.859	0.014	3.312	
68	EUCeSSR479	13	0.829	0.758	-0.098	-0.031	-0.032	0.001	0.801	0.023	3.007	
69	EUCeSSR1042	14	0.200	0.541	0.603	0.677	0.645	0.087	0.723	0.222	2.619	
70	Embra7	28	0.808	0.869	0.073	0.149	0.123	0.029	0.945	0.050	3.710	
71	Embra83	15	0.410	0.611	0.311	0.427	0.410	0.030	0.719	0.130	2.661	
72	EUCeSSR0875	6	1.000	0.671	-0.502	-0.414	-0.446	0.022	0.659	0.000	2.617	
73	Embra369	19	0.733	0.783	0.063	0.180	0.124	0.064	0.873	0.049	3.386	
74	EUCeSSR1087	13	0.718	0.761	0.061	0.116	0.119	-0.003	0.808	0.054	3.098	
75	EUCgSSR21	11	0.672	0.734	0.085	0.164	0.153	0.013	0.802	0.071	2.958	
76	EUCeSSR683	18	0.689	0.811	0.152	0.231	0.203	0.035	0.882	0.076	3.407	
77	EUCeSSR522	16	0.609	0.759	0.212	0.283	0.244	0.050	0.856	0.093	3.265	
78	Embra197[¶]	19	0.869	0.852	-0.020	0.053	0.044	0.010	0.912	0.025	3.521	
79	Embra150	12	0.709	0.669	-0.068	0.047	-0.007	0.054	0.708	0.017	2.767	
80	EUCeSSR0163	22	0.848	0.845	-0.003	0.070	0.051	0.020	0.902	0.031	3.500	**
81	EUCeSSR1070	20	0.593	0.792	0.254	0.355	0.312	0.063	0.907	0.123	3.492	
82	Embra88	29	0.539	0.621	0.117	0.265	0.188	0.096	0.718	0.061	2.790	
83	Embra53[¶]	20	0.491	0.731	0.315	0.453	0.419	0.059	0.841	0.142	3.339	**
84	EUCeSSR0226	5	0.359	0.410	0.098	0.202	0.179	0.028	0.454	0.040	1.945	**
85	EUCeSSR0497	6	0.988	0.684	-0.461	-0.313	-0.400	0.063	0.703	0.000	2.749	

No.[*]	SSR locus	N_A[†]	H_O	H_E	F	F_{IT}	F_{IS}	F_{ST}	PIC[‡]	NAF	A_R	HWE[§]
86	EUCeSSR0845	18	0.613	0.695	0.102	0.225	0.212	0.016	0.780	0.070	2.943	
87	EUCeSSR1125	20	0.734	0.788	0.061	0.160	0.150	0.012	0.834	0.065	3.294	**
88	EUCeSSR0592	10	0.514	0.605	0.166	0.236	0.202	0.042	0.708	0.091	2.585	
89	EUCeSSR0909	28	0.692	0.776	0.103	0.213	0.201	0.015	0.836	0.067	3.329	
90	Embra217	20	0.667	0.803	0.174	0.281	0.233	0.062	0.907	0.091	3.516	**
91	EUCeSSR0679	15	0.278	0.580	0.452	0.615	0.577	0.089	0.743	0.216	2.782	
92	EUCeSSR596	18	0.677	0.818	0.174	0.247	0.226	0.027	0.885	0.097	3.424	
93	Embra40	21	0.519	0.811	0.368	0.410	0.412	-0.003	0.860	0.148	3.490	**
94	EUCeSSR1044	7	0.223	0.253	0.104	0.226	0.168	0.070	0.280	0.031	1.587	
95	EUCeSSR0568	20	0.443	0.772	0.434	0.465	0.464	0.001	0.835	0.193	3.183	**
96	EUCeSSR0126	11	0.409	0.657	0.389	0.445	0.426	0.033	0.759	0.136	2.818	
97	Embra394[¶]	17	0.376	0.609	0.338	0.532	0.449	0.155	0.821	0.134	3.394	**
98	Embra87	27	0.823	0.826	0.001	0.118	0.098	0.023	0.839	0.034	3.631	
99	Embra165[¶]	22	0.793	0.870	0.090	0.152	0.142	0.012	0.927	0.043	3.623	
100	Embra326	17	0.802	0.732	-0.104	-0.016	-0.037	0.020	0.781	0.014	2.984	
101	EUCeSSR1117	16	0.363	0.669	0.456	0.512	0.490	0.044	0.725	0.186	2.814	
102	EUCeSSR0893	14	0.221	0.690	0.680	0.740	0.708	0.109	0.857	0.273	3.233	**
103	Embra269	18	0.659	0.776	0.159	0.258	0.210	0.061	0.866	0.073	3.328	
104	EUCeSSR1021	11	0.697	0.624	-0.127	0.032	-0.015	0.046	0.744	0.018	2.718	
105	EUCeSSR292	11	0.266	0.653	0.565	0.659	0.632	0.077	0.790	0.264	3.000	**
106	EUCeSSR349	20	0.630	0.702	0.109	0.198	0.157	0.048	0.765	0.052	2.963	**
107	EUCeSSR209	6	0.549	0.557	0.030	0.167	0.065	0.109	0.664	0.049	2.487	
108	EUCeSSR0849	13	0.472	0.748	0.351	0.456	0.420	0.064	0.842	0.148	3.321	
109	Embra258	20	0.745	0.802	0.074	0.168	0.134	0.039	0.889	0.045	3.426	
110	EUCeSSR1145	15	0.430	0.534	0.174	0.232	0.240	-0.011	0.609	0.085	2.313	
	Mean	16.9	0.623	0.706	0.108	0.211	0.181	0.037[††]	0.776	0.075	3.027	
	(SE)	(8.2)	(0.006)	(0.004)	(0.008)	(0.022)	(0.022)	(0.003)	(0.150)	(0.070)	(0.532)	

N_A, number of alleles per locus; H_O, observed heterozygosity; H_E, expected heterozygosity; F, fixation index; F_{IT}, inbreeding coefficients of individuals relative to the total population; F_{IS}, inbreeding coefficients of individuals relative to the sub-population; F_{ST}, among-population differentiation; N_M, number of migrants; PIC, polymorphic information content; NAF, null allele frequency; A_R, allelic richness; HWE, Hardy-Weinberg equilibrium.

* Serial number of each SSR locus is the same as that in Supplementary Table S1.

† The total number of alleles for all the 110 loci is 1,857.

‡ There were 103 (93.6 %) highly polymorphic loci according to the criterion of PIC ≥ 0.50 (Botsterin *et al.*, 1980), except the seven (6.4 %) loci EUCeSSR0930, EUCeSSR0705, EUCeSSR231, EUCeSSR0959, EUCeSSR0776, EUCeSSR0226 and EUCeSSR1044.

§ Significant departure from HWE (12 gSSRs and 16 EST-SSRs) over all populations: ** $P < 0.01$. The 12 non-HWE gSSRs (Embra100, Embra98, Embra115, Embra280, Embra377, Embra242, Embra304, Embra187, Embra53, Embra217, Embra40 and Embra394) were excluded from population diversity and structure analyses.

¶ Eight gSSR loci (Embra98, Embra115, Embra280, Embra242, Embra197, Embra53, Embra394 and Embra165; Supplementary Fig. S1) exhibiting F_{ST} outlying values for the 16 populations were excluded from population diversity and structure analyses.

†† The overall mean of F_{ST} for the 31 putatively neutral gSSRs (see Results) is 0.039, which is consistent with between-population comparisons (mean $F_{ST} = 0.037$; Supplementary Table S3) and analysis of molecular variance (AMOVA; among-population variation percentage being 3.7%, $P < 0.001$; Supplementary Table S4).

Supplementary Table S3. Between-population F_{ST} values for 16 *E. grandis* populations based on 31 putatively neutral gSSR loci

Pop. code*	Pic	Cop	MS	Fin	Cre	Kin	Bel	Bor	Con	Kil	MM	MT	ML	Bag	Ora
Cop	0.033														
MS	0.046	0.061													
Fin	0.083	0.081	0.088												
Cre	0.059	0.059	0.074	0.035											
Kin	**0.044**	**0.045**	**0.073**	**0.076**	**0.057**										
Bel	**0.025**	**0.031**	**0.043**	**0.076**	**0.050**	0.012									
Bor	**0.033**	**0.049**	**0.068**	**0.088**	**0.068**	0.004^{NS}	0.011								
Con	**0.029**	**0.040**	**0.041**	**0.063**	**0.046**	0.019	0.003^{NS}	0.013							
Kil	**0.013**	**0.037**	**0.041**	**0.075**	**0.060**	0.023	0.000^{NS}	0.010	0.000^{NS}						
MM	**0.039**	**0.057**	**0.055**	**0.082**	**0.063**	0.008^{NS}	0.018	0.013	0.011	0.014					
MT	**0.028**	**0.039**	**0.057**	**0.063**	**0.050**	0.012	0.004^{NS}	0.015	0.004^{NS}	0.006^{NS}	0.014				
ML	**0.040**	**0.037**	**0.063**	**0.063**	**0.048**	0.034	0.020	0.034	0.020	0.016	0.023	0.020			
Bag	**0.033**	**0.044**	**0.065**	**0.080**	**0.059**	0.028	0.008^{NS}	0.017	0.019	0.015	0.027	0.010	0.031		
Ora	**0.039**	**0.042**	**0.054**	**0.069**	**0.046**	0.022	0.000^{NS}	0.020	0.000^{NS}	0.004^{NS}	0.021	0.004^{NS}	0.026	0.000^{NS}	
Bul	**0.051**	**0.072**	**0.077**	**0.107**	**0.087**	0.024	0.027	0.027	0.011^{NS}	0.021	0.031	0.023	0.057	0.026	0.018

NS, not significant from zero at $P < 0.05$. All F_{ST} values are significant from zero ($P < 0.05$) unless indicated (NS). The overall mean of F_{ST} is 0.037. The bold values are F_{ST} between the northern (codes Pic, Cop, MS, Fin and Cre) and southern (the rest codes) populations (mean F_{ST} = 0.054), while the above are between the northern populations (mean F_{ST} = 0.062) and the right are between the southern populations (mean F_{ST} = 0.016).

* See Table 1 for full description of the populations.

Supplementary Table S4. Analysis of molecular variance (AMOVA) among and within *E. grandis* populations based on 31 neutral gSSR loci

Source	d.f.	SS	MS	Variance component	Percentage of variation
Among populations	15	379.02	25.27	0.49	3.7%
Among individuals within populations	143	2210.27	15.46	2.60	19.4%
Within individuals	159	1632.50	10.27	10.27	76.9%
Total	317	4221.79		13.36	100.0%
F-Statistics	Value	*P*			
F_{ST}	0.037	< 0.001			
F_{IS}	0.202	< 0.001			
F_{IT}	0.231	< 0.001			

d.f., degree of freedom; SS, sum of squares; MS, mean square; F_{ST}, among-population differentiation; F_{IS}, inbreeding coefficients of individuals relative to the sub-population; F_{IT}, inbreeding coefficients of individuals relative to the total population.

References

Acuña, C. V. *et al*. Discovery, validation, and *in silico* functional characterization of EST-SSR markers in *Eucalyptus globulus*. *Tree Genet. Genomes* **8,** 289–301 (2012).

Botstein, D., White, R. L., Skolnick, M. & Davis, R. W. Construction of genetic linkage map in man using restriction fragment length polymorphisms. *Am. J. Human Genet.* **32,** 314–331 (1980).

Brondani, R. P. V., Williams, E. R., Brondani, C. & Grattapaglia, D. A microsatellite-based consensus linkage map for species of *Eucalyptus* and a novel set of 230 microsatellite markers for the genus. *BMC Plant Biology* **6,** 20 (2006).

Faria, D. A., Mamani, E. M. C., Pappas, G. J. & Grattapaglia, D. Genotyping systems for *Eucalyptus* based on tetra-, penta-, and hexanucleotide repeat EST microsatellites and their use for individual fingerprinting and assignment tests. *Tree Genet. Genomes* **7,** 63–77 (2011).

He, X., Li, F., Shi, J. & Gan, S. Seven genomic SSR markers revealed in *Eucalyptus* by re-sequencing of DNA sequences from GenBank. *Silvae Genet.* **60,** 92–94 (2011).

He, X. *et al*. Development of 198 novel EST-derived microsatellites in *Eucalyptus* (Myrtaceae). *Am. J. Bot.* **99,** e134–e148 (2012).

Zhou, C. *et al*. Comparison between direct sequencing and pool-cloning-based sequencing of PCR products in EST-SSR marker development in *Eucalyptus*. *Mol. Plant Breed.* **8,** e1 (2010).

Zhou, C. *et al*. Development of 240 novel EST-SSRs in *Eucalyptus* L'Hérit. *Mol. Breed.* **33,** 221–225 (2014).
